# Automatic change detection: Mismatch negativity and the now-classic Rensink, O’Reagan, and Clark (1997) stimuli

**DOI:** 10.3389/fpsyg.2022.975714

**Published:** 2022-08-25

**Authors:** Domonkos File, Bela Petro, Zsófia Anna Gaál, Nóra Csikós, István Czigler

**Affiliations:** ^1^Institute of Psychology, Eötvös Loránd University, Budapest, Hungary; ^2^Institute of Cognitive Neuroscience and Psychology, Research Centre for Natural Sciences, Budapest, Hungary

**Keywords:** change blindness, visual mismatch negativity (vMMN), flicker paradigm, oddball paradigm, event-related potential (ERP)

## Abstract

Change blindness experiments had demonstrated that detection of significant changes in natural images is extremely difficult when brief blank fields are placed between alternating displays of an original and a modified scene. On the other hand, research on the visual mismatch negativity (vMMN) component of the event-related potentials (ERPs) identified sensitivity to events (deviants) different from the regularity of stimulus sequences (standards), even if the deviant and standard events are non-attended. The present study sought to investigate the apparent controversy between the experience under the change blindness paradigm and the ERP results. To this end, the stimulus of Rensink, O’Reagen, and Clark (1997) was adapted to a passive oddball ERP paradigm to investigate the underlying processing differences between the standard (original) and deviant (altered) stimuli measured in 22 subjects. Posterior negativity within the 280–330 ms latency range emerged as the difference between ERPs elicited by standard and deviant stimuli, identified as visual mismatch negativity (vMMN). These results raise the possibility that change blindness is not based on the lack of detailed visual representations or the deficiency of comparing two representations. However, effective discrimination of the two scene versions requires considerable frequency differences between them.

## Introduction

We often fail to notice considerable changes in our environment even when such changes are inside the focus of our attention. These changes include when the scene is interrupted by a blank field, distracting stimuli, eye blink, or saccadic eye movement, such as by events masking contrast or by movement transience. This effect, called change blindness, was demonstrated by a large body of studies [for review, see Simons et al. (2000) or Jensen et al. (2011)]. Conscious detection of change is a complex cognitive process that involves several sub-processes: (1) representation of the pre-change scene, including those parts of the scene that are outside the focus of attention; (2) maintaining the pre-change representation after the scene has changed; (3) a similar representation of the new scene; (4) the possibility to compare the two scenes and detection of the possible mismatch; and finally, (5) mechanisms of conscious detection of the scene (Rensink, 2002). There is no consensus on which of the above sub-process(es) is/are responsible for the robust phenomenon of change blindness. From change blindness studies (e.g., Rensink et al., 1997), we know that the final stage of information processing (conscious detection) is often not reached, but results are equivocal about at what stage of processing unattended information terminates. On one extreme, it is assumed that the visual system does not establish detailed representation about the non-attended parts of scenes (O’Regan and Noe, 2001). Concerning memory maintenance, the following theories are prevalent: detailed representations are superimposed by subsequent input (Landman et al., 2003); or even if the representations are available, the comparison process is absent (Simons et al., 2002; Mitroff et al., 2004); or else, the change is automatically detected, but there is no conscious representation (e.g., participants cannot report) of the change (Fernandez-Duque and Thornton, 2000).

The goal of the present study was to contribute to this issue. To this end, we investigated the possibility that whether stimuli used in a classical demonstration of change blindness (Rensink et al., 1997) elicit the visual mismatch negativity (vMMN) component of event-related potentials (ERPs), if these stimuli are presented in a passive oddball paradigm, deviated as little as possible from the original change blindness paradigm. VMMN emerges to visual events that are different from the representation of regular stimuli within a sequence, even if those visual events are unattended and completely unrelated to an ongoing task. The advantage of using this measure is that it allows one to gather information about processing of non-attended events, even in the absence of conscious detection.

Visual mismatch negativity is usually investigated in the passive oddball paradigm, in which participants perform a visual (or sometimes auditory) task, while the vMMN-related events are presented outside the context of the task, as unattended stimuli. VMMN is a negative ERP difference component of unattended, sequentially presented regular visual stimuli (standards) and stimuli that violate the regularities of this stimulus sequence (deviants), measured at posterior electrode locations at approximately 100–350 ms after stimulus onset (for review, see Kimura et al., 2011; Stefanics et al., 2014). The process underlying the emergence of vMMN is considered an automatic adjustment of predicted and incoming events; in the case of a difference between the two, vMMN emerges. VMMN is elicited not only by simple deviant features (e.g., color, spatial frequency, and orientation) but also by deviancies of higher order features, like visual categories, facial emotions, gender, and age of portraits (for review, see Kimura et al., 2011; Stefanics et al., 2014).

Event-related potential studies investigating change detection frequently applied the “single-shot” paradigm, where stimulus pairs are presented, and participants decide whether the stimuli are the same or different. Using this paradigm, Kimura et al. (2008) presented different colored dot patterns surrounding the field of the primary task (a simple detection task). In change trials, the color of a dot changed, and the task was to indicate whether the pair of dots were the same or different. Comparing ERPs to non-detected color change and no change, an anterior positivity emerged in the 160–180 ms range. Ball et al. (2015) presented pairs of stimuli containing eight objects. Between the pairs, mudsplashes caused change blindness effects. In change trials, one of the objects was semantically related or unrelated. The change detection task was secondary, and the primary task was to detect the orientation change of letters. Under change blindness (i.e., in trials with non-reported object change), a late negative difference potential (1,530–1,730 ms range) emerged as the difference between unrelated and related object changes. The authors localized the negativity into left inferior, left middle occipital, and middle temporal areas. These results show the possibility of unreported change detection, but it is doubtful that such late effect is due to the perceptual activity involved in change detection.

In several studies, no differences were observed between non-detected change and no-change stimuli. Niedeggen et al. (2001) obtained ERP differences using alphanumeric stimuli, showing identity of localization change only for detection trials and other trials immediately before detection trials. In a single alternation design with orientation change of rectangles, there was an ERP difference between non-detected change and no-change trials (Koivisto and Revonsuo, 2003). Henderson and Orbach (2006) presented pairs of patterns consisting of grating patches. In a “same–different” task, they obtained no significant ERP effects for non-detected changes. In an S1–S2 paradigm (Schankin and Wascher, 2007), a letter identification task was combined with a change detection task, and this was introduced as a change in one of the dots within a dot pattern. No ERP differences were obtained when comparing non-detected change with no-change trials.

In a study (Fernandez-Duque et al., 2003) with multiple stimulus presentations, everyday scenes were shown for 12–22 repetitions before the scene was changed. Due to the considerable number of cycles appearing before the detected change, it was possible to record ERPs to non-detected changes (“unaware changes”). When comparing the no-change to non-detected changes, there was an anterior deflection in the 240–300 ms range.

Lyyra et al. (2012) presented oddball sequences of everyday color photographs, where the rare (deviant) stimuli were variants of the frequent ones (standards). Participants were instructed to search for a change in the images and to report it by pressing a button when they first noticed the change. The authors calculated differences between the activity in response to non-reported changes (two changes before the reported one) and no-change stimuli before such non-reported changes. At short (100 ms) inter-stimulus intervals, they reported a negative difference potential within the 200–260 ms range over the posterior locations. The authors interpreted this finding as an emergence of the visual mismatch negativity (vMMN) ERP component. It is important to note that the average number of changes required for behavioral detection was 10.3. This number is similar to the repetition number in the traditional change blindness studies, showing that the change detection process in the oddball paradigm is similar to that of traditional change blindness paradigms. In an earlier study (Lyyra et al., 2010) with similar methods, ERP differences were reported to non-detected change versus no change as early as 60–100 ms after the stimulus onset, with positive polarity, at anterior locations. In a single alternation (S1–S2) paradigm using facial stimuli, Eimer and Mazza (2005) reported the onset of an ERP effect of non-detected change with a similar latency. However, the authors argued that this effect was due to a preparation difference between the trials, which was supported by a second experiment.

No-report versus no-change responses were compared in the time domain in a “same–different” (S1–S2) task (Darriba et al., 2012). The stimuli were sinusoidal gratings that either did not change or the orientation changed. After S2, the power of anterior beta band was weaker in the non-detected change trials than in the no-change trials. This difference appeared as early as 118–180 ms post-stimulus.

In an fMRI study (Beck et al., 2001), participants were asked to detect changes in either of two peripherally presented visual images while simultaneously engaging in a primary letter detection task. Four stimuli–either faces or outdoor scenes–were presented, allowing for three possible changes within a trial. Comparing non-reported change trials and no-change trials in the face condition, activation was obtained only in the fusiform gyrus, lingual gyrus, and inferior frontal gyrus. This pattern of activation was markedly different from the activation of the wide networks activated in case of detected changes. However, in ERP analyses, they obtained no difference between the two conditions. Although it is difficult to attribute these results to any specific cognitive operation, it shows that the processing system detected the stimulus change, even if the participants did not report them.

In the current study, our aim was to investigate whether vMMN appears for changes in natural scenes for which we have behavioral data that conscious detection of changes is difficult. The relevance to investigate such question is that our knowledge of vMMN elicited by natural images is relatively restricted since most studies employ single objects, or a set of simplistic geometric shapes. Even in studies using complex scenes (e.g., Lyyra et al., 2012), the task was the active search for changes. Also, no vMMN study was conducted with a stimulus set, from which detailed behavioral data are available. As a consequence, our knowledge about the relation between conscious (reflected in behavioral performance) and the pre-attentive (reflected in vMMN) processes is limited. Thus, our secondary aim was to explore whether vMMN is sensitive to the semantic-level characteristics of natural images, from which Rensink et al’s (1997) study demonstrated that conscious perception is highly sensitive. Change blindness studies had demonstrated that even significant changes remain unnoticed, if those changes are unattended. Thus, passive stimulus presentation using image pairs from change blindness experiments models the event when the change in the altered image is not attended and consequently not noticed. If vMMN is elicited in such condition, it would indicate (1) representation of the pre-change scene, including those parts of the scene that are outside the focus of attention; (2) maintaining the pre-change representation after the scene has changed; (3) a representation of the new scene; and (4) the possibility to compare the two scenes and detection of the possible mismatch. We hypothesized that this set of processes are active only if the change violates the sequential regularity of the stimulus sequences. In other words, in order to record the ERP signature of automatic visual change detection, it is necessary to establish a memory representation of the regular scenes.

## Materials and methods

### Participants

A total of 22 volunteers (17 women; mean age: 21.95 years, SD = 2.78) participated in the study for compensatory payment. They had no ophthalmologic or neurological abnormalities. Written informed consent was obtained from all of the participants prior to the experimental procedure. The study was conducted in accordance with the Declaration of Helsinki and was approved by the Joint Committee of Ethics of the Psychology Institutes, Hungary.

### Stimuli and experimental design

**FIGURE 1:**
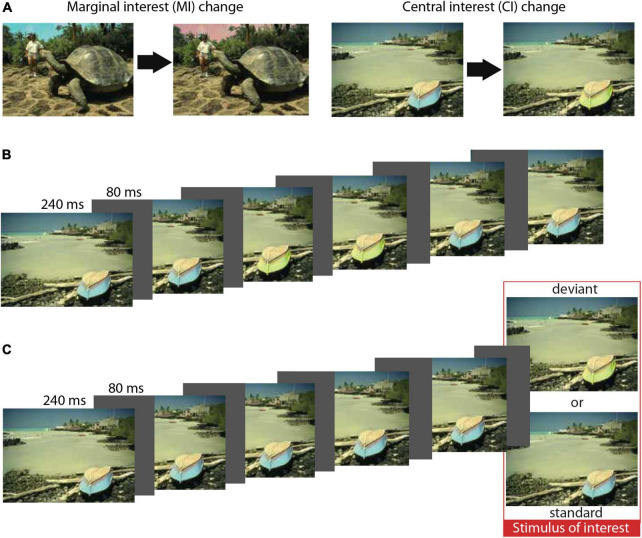
Conditions and sequences. **(A)** An example for central and marginal conditions (color change). **(B)** Illustration of the original change blindness paradigm. **(C)** Illustration of the oddball sequence used in the current study. Reproduced with permission from Ronald Rensink.

We selected the stimuli from the same set of 48 color images previously used in the experiment of Rensink et al. (1997). The selected sets encompassed a considerable variety of changes: color, location, and absence of a particular object. All changes were categorized as central interest (central) or marginal interest (marginal), as identified in the original study (Rensink et al., 1997). It was determined via an independent experiment in which five participants provided a brief verbal description of each image: Central interests were defined as objects or areas mentioned by three or more participants; marginal interests were objects or areas mentioned by none. For an example, see Figure 1A. Importantly, physical parameters were balanced between the two groups of images such that the average change in intensity and color of marginal and central was similar, while the areas of the marginal changes (mean difference = 22 square degree) were somewhat larger than those of the central changes (mean difference = 18 square degree) (Rensink et al., 1997).

In the present study, images were presented in sequences with the same timing as in the original experiment; thus, each image was displayed for 240 ms with an 80-ms-long blank interval {gray [RGB (84,83,83)] screen, see Figure 1 for illustration} between them. In the present variation of the oddball paradigm, an image sequence consisted of 5–8 standards, followed by the stimulus of interest, which was either a standard (i.e., the same picture as previously presented) or a deviant (i.e., the changed version of the image) (see Figure 1C). Each stimulus of interest was followed by a 700-ms blank interval {gray [RGB (84,83,83)] screen, see Figure 1 for illustration}.

Each condition (color-marginal, color-central, location-marginal, location-central, absence-marginal, absence-central) contained four set of scenes, each presented in 20–20 standard and deviant stimulus trains; thus, 80 standards of interest and 80 deviants were presented for each of the six conditions. The total of 960 stimulus trains [6 (conditions) × 2 (standard, deviant) × 4 (scene) × 20 (repetition number for each scene)] were presented in 10 blocks. Stimulus trains of different conditions and images followed each other in a semi-random order within a block, with the rule that two trains of the same condition being never displayed one after the other. In the absence condition, a particular object was absent from the deviant stimuli; in the color condition, the color of a particular object changed, while in the location object, the position of an object changed.

### Task

Participants performed a simple reaction time task independent of the stimulus presentation. A cross was displayed at the center of the screen, which comprised a shorter (0.34°) and a longer line (0.68°), and a button press was required for each reversal of the size of the lines. The cross changed randomly between 5 and 15 s, and the participants were asked to respond as quickly and as accurately as possible. This task required central fixation, which prevents scanning of the stimulus.

### Recording and measuring the electrical brain activity

The electroencephalographic (EEG) activity was recorded (DC-70 Hz; sampling rate, 1,000 Hz; BrainVision Recorder 1.21.0303, ActiChamp amplifier), with active electrodes placed at 32 locations according to the extended 10–20 system, using an elastic electrode cap (EasyCap, Brain Products GmbH). The online reference electrode was at FCz, and then, the activity was re-referenced offline to the electrode on the nose tip. Horizontal electrooculographic activity was recorded with a bipolar configuration between the electrodes that were positioned lateral to the outer canthi of the eyes. Vertical eye movement was monitored with a bipolar montage between the electrodes that were placed above and below the right eye. The impedance of the electrodes was kept below 10 kΩ.

EEG signals were filtered offline (0.1–30 Hz, 24 dB slope). Epochs of 600 ms, starting from 100 ms before the stimulus onset, were averaged separately for the standard and deviant stimuli. Trials with an amplitude change that exceeded ± 100 μV on any channel were rejected from further analysis. Only the responses elicited by the stimuli of interest (see stimuli and experimental design and Figure 1C) were included into the standard and deviant-related average ERPs. Responses were averaged separately for stimulation type (standard, deviant), deviation type (color, location, absence), and interest (marginal, central), for example, standard–color–marginal. The mean number of accepted trials for each ERPs within a subject was given as follows: 64 (SD = 5.5) for color–marginal standard, 62.6 (SD = 5.5) for color–marginal deviant, 63.1 (SD = 7.2) for color–central standard, 62.5 (SD = 7) for color–central deviant, 62.9 (SD = 4.7) for location–marginal standard, 61.9 (SD = 6.7) for location–marginal deviant, 63 (SD = 6) for location–central standard, 62.6 (SD = 6.83) for location–central deviant, 62 (SD = 7.1) for absence–marginal standard, 61.9 (SD = 6.42) for absence–marginal deviant, 63.3 (SD = 5.7) for absence–central standard, and 62.9 (SD = 6.2) for absence–central deviant. Difference potentials were formulated as the difference of the standard and the deviant of the same deviation type and interest; color–marginal, color–central, location–marginal, location–central, absence–marginal, and absence–central.

Based on previous vMMN studies (Kimura et al., 2009; Sulykos and Czigler, 2014; File et al., 2017), we expected the emergence of a deviant–minus–standard difference wave over the posterior electrode locations. To reinforce this expectation, we defined a 2 × 3 matrix of electrodes (PO3, POz, PO4, O1, Oz, O2), based on previous studies (e.g., File et al., 2017, 2020; Yan et al., 2017). To measure vMMN, we adopted the method suggested by Luck and Gaspelin (2017) (see also Ford et al., 2022). According to the method, ERPs to all standards and deviants were collapsed across the six types of scenes on the 2 × 3 matrix of electrodes (PO3, POz, PO4, O1, Oz, O2), and a difference wave vMMN was calculated by subtracting the collapsed standards from the collapsed deviants (“collapsed difference”). On the collapsed difference potential, a point-by-point *t*-test was applied against zero (*p* < 0.05), and VMMN was identified as a negative deflection of at least 20 consecutive data points. The 280–330 ms section met this criterion. In the first step, an omnibus ANOVA was calculated within the 280–330 range [factors of scene (color, location, absence), interest (marginal, central), deviance (deviant, standard), laterality (left, medial, right), and anteriority (parieto-occipital, occipital)]. Thereafter, in separate ANOVAs, we investigated the six scene types [(color, location, absence) × (marginal, central)] separately. Due to the earlier emergence of the negativity in the location–central condition, with an exploratory intent, we also calculated a similar ANOVA in the 250–300 ms range, in a range well beyond the range of the “collapsed difference.” In *post hoc* comparisons, the Bonferroni correction was used. When appropriate, we applied the Greenhouse–Geisser correction. Effect sizes were presented as η*_*p*_^2^*.

## Results

### Behavioral results

The participants performed the primary task with hit rates over 80% (mean hit rate = 95.8%, *SD* = 4.1), which indicated active attention on the task. The mean RT was 552.3 ms (*SD* = 83.3). There was no difference in performance between the conditions.

### Event-related potentials

**FIGURE 2:**
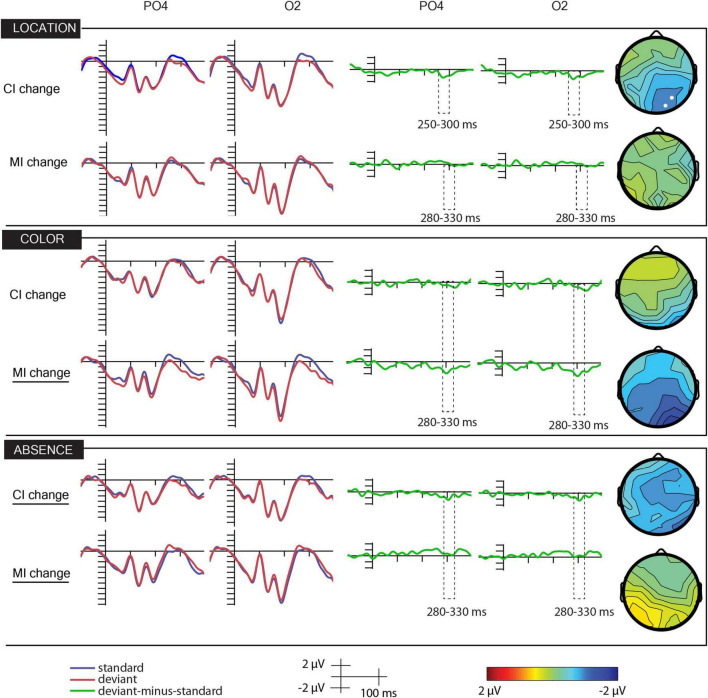
Grand-averaged event-related (first two columns from the left), difference (third and fourth columns from the left) potentials, and scalp distributions of the differences (fifth column from the left) of the location, color, and absence scenes of conditions marginal and central change.

Figure 2 shows the ERPs to deviant and standard stimuli, the deviant-*minus*-standard difference potentials, and the surface distribution of the difference potentials within the 280–330 ms latency range. As the figure shows, in this range, the difference potentials indicated negative deflections. Table 1 indicates the amplitude values in the PO2 and O2 locations in the 280–330 ms time range.

**TABLE 1:** Mean amplitudes of the ERPs (in brackets, the standard deviation) to the standards and deviants (μV) at the PO4 and O2 locations in the three kind of scenes (color, location, absence) at marginal and central changes.

		Marginal		Central	
		Standard	Deviant	Standard	Deviant
Color	PO4	−0.04 (0.34)	−1.57 (0.45)	−0.22 (0.36)	−0.80 (0.42)
	O2	0.23 (0.36)	−1.52 (049)	0.04 (0.379	−0.71 (0.44)
Location	PO4	−0.57 (0.37)	−0.54 (0.28)	0.85 (0.49)	−0.15 (0.28)
	O2	−0.33 (0.38)	−0.17 (0.27)	1.10 (0.40)	0.06 (0.39)
Absence	PO4	−0.22 (0.41)	0.05 (0.31)	0.06 (0.39)	−0.84 (0.41)
	O2	−0.12 (0.41)	0.05 (0.32)	0.32 (0.37)	−0.51 (0.40)

The measurement range is 280–330 ms, except the location–central condition, where the range is 250–300 ms.

Most importantly, according to the ANOVA with factors of scene (color, location, absence), interest (marginal, central), deviance (deviant, standard), laterality (left, medial, right), and anteriority (parieto-occipital, occipital). We obtained a significant effect of deviance, *F*(1,21) = 7.41, *p* < 0.05, η*_*p*_^2^* = 0.26, indicating a higher amplitude response to deviant stimuli (−0.57 vs. −0.01 μV). The main effect of anteriority was also significant, *F*(1,21) = 24.7, *p* = 0.01, η*_*p*_^2^* = 0.27. ERPs had larger negativity at PO locations (−0.45 vs. −0.2 μV). The significant deviance × laterality interaction, *F*(2,42) = 10, 45, *p* < 0.001, ε = 0.62, η*_*p*_^2^* = 0.33, was due to the smaller deviant–standard difference at the left side, but according to the *post hoc* Bonferroni correction, even at the left side, the difference was significant. However, these effects were qualified by higher order interactions, involving the scene and interest factors, that is, scene × deviance × laterality, *F*(4,84) = 2.73, *p* < 0.05, ε = 0.69, η*_*p*_^2^* = 0.12, and scene × deviance × interest × laterality, *F*(4,84) = 2.72, *p* < 0.05, ε = 0.52, η*_*p*_^2^* = 0.12.

Due to these differences, we calculated separate ANOVAs to the six conditions of scenes (color, location, absence, and marginal and central) in each case. The factors were deviance, laterality, and anteriority.

In the color–marginal scenes, we obtained significant effect of deviance, *F*(1,21) = 12.25, *p* < 0.001, η*_*p*_^2^* = 0.38. The anteriority main effect, *F*(1,21) = 5.21, *p* < 0.05, 0.20, and deviance × laterality interaction, *F*(2,42) = 12,10, *p* < 0.0001, ε = 0.73, η*_*p*_^2^* = 0.37, were also significant. ERPs had higher amplitude at the occipital locations, and deviant stimuli elicited larger negativity than the standard ones. Concerning the interaction, according to the Bonferroni correction, deviant-related negativity emerged at all levels of the laterality factor, whereas there was no difference between the amplitudes of the ERPs to the standard, and deviants at the right side elicited larger negativity than those at the left side.

In the color–central scenes, the deviance × anteriority interaction was significant, *F*(1,21) = 4.81, *p* < 0.05, η*_*p*_^2^* = 0.19. According to the Bonferroni correction, deviant stimuli elicited larger negativity at the occipital locations, and the difference was larger occipitally.

In the location–marginal scenes, the main effect of anteriority, *F*(1,21) = 6.80, *p* < 0.05, η*_*p*_^2^* = 0.24, and the deviance × location interaction, *F*(2,42) = 3,57, *p* < 0.05, ε = 0.78, η*_*p*_^2^* = 0.15, were significant. The anteriority main effect was due to the larger negativity of ERPs at the parieto-occipital sites. Concerning the interaction, in the midline, deviants elicited larger negativity, but according to the Bonferroni correction, the difference was non-significant.

In the location–central scenes, the main effect of anteriority, *F*(1,21) = 14.44, *p* < 0.01, η*_*p*_^2^* = 0.41, and the laterality × anteriority interaction, *F*(2,42) = 3,26, *p* < 0.05, η*_*p*_^2^* = 0.13, were significant. ERPs were more negative at the parieto-occipital locations. According to the Bonferroni correction, ERPs in the midline were more positive than those at the right side. However, in this condition, the negativity emerged earlier. Therefore, we conducted a similar ANOVA on the 250–300 ms range. In this range, the anteriority main effect was significant, *F*(1,42) = 3,57, *p* < 0.05, ε = 0.78, η*_*p*_^2^* = 0.15. Positivity was larger at the occipital locations. The deviance × laterality interaction was also significant, *F*(2,42) = 3,94, *p* < 0.05, ε = 0.78, η*_*p*_^2^* = 0.16. According to the Bonferroni correction, ERPs to deviants had smaller amplitude (i.e., the deviant-minus-standard wave showed a negative deflection) in all factors of laterality. In the location–marginal scenes, there were no ERP differences between the standard and the deviants in the 250–300 ms range.

In the absence–marginal scenes, the deviance × laterality interaction was significant, *F*(2,21) = 10.47, *p* < 0.01, η*_*p*_^2^* = 0.33. According to the Bonferroni correction, deviants elicited *smaller negativity* at the left side and in the midline.

In the absence–central scenes, we obtained significant anteriority, *F*(1,21) = 7.07, *p* = 0.01, η*_*p*_^2^* = 0.25, and laterality main effects, *F*(2,42) = 4.86, *p* = 0.01, ε = 0.97, η*_*p*_^2^* = 0.19. The main effect of deviance approaches significance, *F*(1,21) = 3.33, *p* < 0.08, η*_*p*_^2^* = 0.14. ERPs were more negative in the parieto-occipital locations and more negative at the left than on the right side. The main amplitude of the standard stimuli was 0.07 μV, and that of the deviants was −0.72 μV.

## Discussion

The current study aims to investigate whether the electrophysiological signature of automatic change detection, the vMMN component, of ERPs is sensitive for changes in natural scenes, for which we have behavioral data showing conscious detection of changes is difficult (Rensink et al., 1997). A secondary aim was to test whether vMMN, if elicited by such changes, reflects differences at the semantic level. To this end, we adapted the stimuli of Rensink et al’s (1997) study, which resulted in robust change blindness (detection required 7.3 stimulus in sequences with changes of central changes and 17.1 cycles along with changes of marginal interest) to a vMMN paradigm. The current paradigm has two characteristic features: (1) the stimuli are task-irrelevant, and there is no instruction to deal with these stimuli; (2) one of the versions of the stimuli is frequently presented (standard), whereas the other is rare (deviant). We obtained that within such non-attended sequences of complex pictures (scenes), the rare (deviant) versions of scenes are capable of eliciting vMMN, that is, a posterior negativity within the 250–330 ms range. It is important to emphasize that vMMN signifies a specific type of change. This ERP component is elicited when the stimulus change violates the regularity of stimulus sequences (Kimura et al., 2011; Stefanics et al., 2014). Discussion of the contrast between the change blindness results and the present vMMN results is needed.

Concerning the possibility of automatic buildup of detailed visual representation, the present results raise the possibility that change blindness is not based on the lack of detailed visual representations (O’Regan and Noe, 2001) or the deficiency of comparing two representations (Simons et al., 2002; Mitroff et al., 2004). It is important to note that there were two fundamental differences between a typical change blindness paradigm and our study; therefore, this interpretation needs further evaluation and the consideration of alternative explanations. The first difference was the stimulus presentation: while in the original study, image pairs were presented in an alternating sequence (AAA′A′, where A is the original image and A′ is the modified image, see Figure 1B), in the current study, the modified image was presented after the repeated presentation of the original image (AAAAAA′, see Figure 1C). To generalize the interpretation of the current study to the original change blindness results, we argue that in order to measure vMMN, a sequential rule must be formed beforehand, which can then be violated. For that, images of the oddball sequence must be compared in order to classify the incoming stimulus as a standard (which further strengthens the representation) or as a deviant (which violates the rule). Since we applied the identical stimulus duration and inter-stimulus interval as in the original, Rensink et al. (1997), study it is reasonable to assume that a similar comparison process took place in the change blindness paradigm. If our reasoning is correct, it is reasonable to assume that the change is automatically detected, but there is no conscious representation (e.g., participants cannot report) of the change, in accordance with Fernandez-Duque and Thornton (2000). One might argue that results of an oddball sequence are not comparable to results of a flicker paradigm. Considering that in the original sequence, each image was presented twice before being switched, and the difference between the two sequences is rather qualitative than quantitative. This reasoning is supported by the results of Lyyra et al. (2012) reporting the number of changes required for behavioral detection of change within an oddball paradigm is very similar to the ones reported for the flicker paradigm. Also, based on Rensink et al. (1997) reasoning, repeating the same image creates a temporal uncertainty as to when the change is being made, thus noticing changes within an oddball sequence is potentially more difficult.

The second difference was that the current paradigm did not require conscious change detection. In the vast majority of vMMN studies, the authors presumed that the task-irrelevant changes were outside the scope of consciousness. This is a reasonable assumption in studies having proper control of the relationship between the task-relevant and vMMN-related events. However, relatively few studies have directly investigated whether vMMN was really elicited by deviants which were undetected at the conscious level. In active oddball paradigms (where the participants searched for changes), Lyyra et al. (2010, 2012) observed change-related ERP differences to non-detected changes. Chen et al. (2020) reported vMMN in response to masked fearful emotional faces, even if the participants did not report seeing the facial stimuli. Using simple stimuli (grid pattern), Czigler and Pató (2009) found vMMN to deviant grid orientation unnoticed by the participants. In the current study, no behavioral data were collected on change detection performance, which should be considered as a limitation. However, we argue that in the current study, it is reasonable to assume that changes were not detected consciously; based on that, in the original study, 7–17 cycles were necessary for change detection (even though actively attended), while in the current study, only one stimulus change occurred after 5–8 presentations of the original image. Furthermore, in other vMMN studies using the same control task for attention in which the change between images is clear if attended (e.g., Kecskés-Kovács et al., 2013), participants usually report a feeling that something was changing in the background, but explicit descriptions are rare. However, a simple questionnaire or behavioral test would have been useful in the current study and would be beneficial to adopt as a part of the vMMN research protocol in general.

We have to emphasize that despite the main effect of deviance (i.e., emergence of vMMN) in the omnibus ANOVA, reliable vMMN appeared only in the color–marginal, color–central, and location–central conditions, and there were some hints of vMMN in the absence–central condition. Deviant color change elicited vMMN even in the case where in Rensink et al.’s (1997) study, this change was marginal; thus, it seems that from the changing dimensions, color is stronger than the other two investigated factors. This assumption is supported by the results of the original study, in which the smallest difference between marginal and central conditions in the required alternations for change detection was in the color change condition. In the location scenes, only changes of central interest elicited vMMN, and in the absence scenes, the tendency of vMMN appeared also only in case of central interest. This way, the present results partially supported the distinction by Rensink et al. (1997). An interesting related topic covers the role of top–down predictions in change detection. From experiments investigating change detection performance for subjectively high salience changes, a growing number of evidence suggests that attentional biases highly affect behavioral performance. For example, problem drinkers detect alcohol-related changes presented in a flicker paradigm with shorter latency than social drinkers (Jones et al., 2006). Also, fear-relevant stimuli (snakes) were detected with a shorter latency than natural changes, and this effect was facilitated by the subjective level of fear from snakes (Rosa et al., 2011). A similar attentional bias has been reported for vMMN; internet-related deviances elicited higher vMMN for internet addicts than for healthy controls (He et al., 2018). Although the current results only partially support the possibility of a pre-attentive scene context analyses embedded in the process of automatic change detection, further studies investigating the flicker paradigm and the current modified oddball paradigm might reveal the role of vMMN in directing attention to relevant changes.

In our study, vMMN dominated at the right side. This difference in color deviance is not unprecedented (e.g., Zhong et al., 2014). For location deviance, we obtained no previous data.

Since the ERP difference between the effects of the deviant and standard stimuli could be the result of either a decrease in activity in response to the standards (Krekelberg et al., 2006; May and Tiitinen, 2010; May, 2021) or additional activity elicited by the deviants (Kimura et al., 2011; Stefanics et al., 2014), an important limitation of the current study is the lack of control sequences. The initial reasoning for not applying control sequences was based on the physical differences between marginal and central interest changes, that is, the areas of the marginal changes (mean difference = 22 square degree) were somewhat larger than the those of central changes (mean difference = 18 square degree) (Rensink et al., 1997). Since the amplitude of ERP responses reflecting adaptation is sensitive for physical parameters, higher amplitude standard–deviant differences for marginal changes (compared to central changes) would indicate adaptation-based cognitive processes, with Occam’s razor in mind. However, higher amplitude standard–deviant differences for central changes would indicate a semantic-level representation (on top of a very likely adaptation effect), which potentially reflects vMMN (considering the paradigm, topography, and latency). Since the current results were not consequent in this respect, the presence of “genuine-vMMN” (e.g., see Kimura et al., 2009; File et al., 2017) cannot be established with certainty. Another indicator to differentiate between adaptation and “genuine vMMN”-related processes is the latency of the difference signal. Kimura et al. (2009) described the 100–150 range to reflect adaptation, while “genuine vMMN” appeared later, in the 150–350 range. It is important to note however that stimuli in the current study (natural images) were very different from the stimuli of the Kimura study (oblique lines); thus, direct translation of those results would not be wise. Discussion on theories about the brain structures necessary for conscious detection (“neural correlates of consciousness”), as well as theories of various levels of consciousness (Block, 2005; Chen et al., 2020), are beyond the scope of the present study, but in light of the present results, this topic deserves a short discussion. As a general view, conscious detection requires recurrent activation of various posterior and anterior structures (e.g., Lamme, 2006). Concerning the change detection studies (after several cycles of the alternative scene variants, eventually there is a valid report of the locus and identity of the change), the report requires spatial–visual attention (Rensink et al., 1997). The question we examined is whether the mechanisms underlying vMMN contribute to such attentional processes, for instance, these processes may influence the saliency map about the scene (Wolfe, 2007), or alternatively, to turn this question around: what is the exact function underlying vMMN in the sequence of information processing. The predictive coding theory of (auditory and visual) mismatch negativity (Garrido et al., 2008; Stefanics et al., 2014) claims that the function of the automatic processes underlying the mismatch responses is to adjust the expected pattern of activity to the actual pattern of activity. In cases of instantaneous matches, when several presentations of the input are identical, the brain system spares energy (i.e., no further processing is needed), whereas in cases of mismatch, adjustment requires multi-stage processing. The oddball paradigm is the model for such a scenario. Accordingly, in this framework, the processes underlying the (auditory and visual) MMN are confined to the perceptual system (stimulus identification). However, as Näätänen originally proposed (Näätänen et al., 1978), a function of processes underlying MMN is to initialize orientation reaction; therefore, mismatch may contribute to the involvement of the attentional system. We argue that the scenario of the change blindness paradigm is different from the scenario of the oddball paradigm because the probability to build a predictive representation of a particular stimulus variation is lower. It seems that in order to detect the difference in the change blindness situation, we need to use the mechanisms of spatial attention and the limited storage capacity working memory (e.g., Cowan, 2010). As a consequence, to scan the scenes, several presentation cycles of the alternative scenes are needed.

In summary, our findings indicated that the cognitive system of visual perception is able to detect changes in a passive oddball paradigm applying image pairs of natural images. This result suggests that the difficulty in reporting such changes in the flicker paradigm may not be due to a lack of detailed visual representation or a failure to compare the two representations but rather probably because of a failure to build a model for one of the commonly presented stimuli. In order to detect the difference, processes of spatial attention are needed. By contrast, in biased presentation of the two versions, the frequently presented one is capable of acquiring a visual representation that is sensitive to changes of the scene.

## Data availability statement

The raw data supporting the conclusions of this article will be made available by the authors, without undue reservation.

## Ethics statement

The studies involving human participants were reviewed and approved by the Joint Committee of Ethics of the Psychology Institutes, in Hungary. The patients/participants provided their written informed consent to participate in this study.

## Author contributions

IC supervised the project, performed the calculations, and wrote the manuscript with the support of DF. BP planned and programmed the stimulation, analyzed the behavior data, and organized the experiments. NC carried out the experiments. ZG composed the text. DF took part in the development of the experiment and data processing and analysis. All authors contributed to the article and approved the submitted version.
